# The circulation analysis of substandard foods in China based on GIS and social network analysis

**DOI:** 10.1371/journal.pone.0248037

**Published:** 2021-03-05

**Authors:** Yakun He, Jiadong Jiang, Shuo Li

**Affiliations:** China Aero-Polytechnology Establishment, Key Laboratory of Quality Infrastructure Efficacy Research, SAMR, Beijing, China; Institute for Advanced Sustainability Studies, GERMANY

## Abstract

In China, the majority of food enterprises are small-sized and medium-sized. While the supervision costs are high, food safety issues are still emerging. Food circulation is an indispensable part in the entire food chain. At present, there are few studies on the regional spread of food safety risks in the circulation field from a macro perspective. This study combines GIS and social network analysis methods to deeply explore the regional circulation characteristics of substandard foods. First, we crawl the dataset of Food Safety Sampling Inspection Result Query System. Then we obtain the geographical locations of the manufacturers and distributors by GIS. Finally, we construct the province-level and city-level substandard foods’ circulation networks, and employ social network analysis to target key cities and paths. The experimental results show that the circulations of substandard foods are characterized by dense province-level network and sparse city-level network, and they are mostly local and short-distance trafficking. 361 cities are divided into 13 city clusters considering the network connection characteristics. Chongqing, Beijing, Zhengzhou, and Changsha are identified as key cities by all measurement indicators, and at least four indicators can identify Shanghai and Wuhan. These cities have the highest priority for combating substandard foods’ circulation networks.

## 1. Introduction

China is the developing country who has an enormous population and great geographical heterogeneity. During the past few decades, China has made a leap from food shortage to basically eradicating hunger. However, there are obvious conflicts between traditional agriculture and numerous food enterprises, and consumers’ demands for safe and high-quality foods are becoming stronger. A major difference between China’s food industry and that of developed countries is the scale of operations. More than 69% of China’s food companies are small-sized and medium-sized enterprises, which produce most of foods for the country [1]. China is in the transitional period of transition to developed country and is facing many deliberate food adulteration issues and food fraud issues. Many companies are still driven by the attitude of "fast profit is above everything", especially small-sized and medium-sized enterprises whose profits are so low that they would not afford additional costs to ensure their products’ quality. Therefore, the supervision is more complicated and more difficult to perform than other countries. Food safety supervision is the joint responsibilities of national, provincial and local government departments. At the same time, the food industry (including production, processing, sales and service industries) bears the major responsibilities for providing consumers with safe foods [2, 3].

Food safety supervision and inspection began in 1949 when the People’s Republic of China was founded, and it has always been an important part of national laws and regulations. Its development has gone through several important periods, including technical management without government power (disease prevention and quarantine), administrative supervision (Ministry of Health), multi-government agency supervision and single government agency supervision [4–6].

The state organizes food safety sampling inspections every year, and has accumulated a big and authoritative food sampling quality and safety dataset. In recent years, the pass rate of food sampling inspections has been climbing from 95.4% in 2014 [7] to 97.6% in 2019 [8]. However, food safety issues still exist in fragments and have certain geographical distribution characteristics, which needs refined analysis and processing. Small-sized and medium-sized food enterprises are usually distributed in rural areas, sometimes even beyond the supervision of governments. Therefore, geographical spatial differences need to be considered for refined analysis in food production and sales analysis [9]. At the same time, the government can cut off the sales chain of counterfeit and inferior foods through refined supervision, so that related small enterprises have no profit to be made. They will take the initiative to expand their scale through mergers to achieve economies of scale. In this way, the proportion of small enterprises will be greatly reduced, and food safety will also be improved.

The existing researches on food quality and safety in China mainly include the following four aspects: (1) Food-borne diseases: food-borne pathogenic bacteria [10, 11], food-borne mycotoxins [12, 13], food-borne viruses [14, 15], food-borne parasitic diseases [16, 17]; (2) Food chemistry: food additives [18], pesticide and veterinary drug residues [19], heavy metal contamination [20], food fraud [21, 22]; (3)Risk assessment and communication: microbiological risk assessment in food [23, 24]; (4) Risk management: food safety laws and regulations [25], food safety standards [26], food safety regulatory inspection [27]. The existing researches mainly focus on the micro analysis of physical and chemical characteristics of the specific food, the risk analysis of the specific food’s supply chain at the mesoscale, and the macro policy analysis of food safety and law. At present, there are few studies on the regional dissemination of food safety risks from a macro perspective.

Spatial data is a very important type of data. About 80% of the data in daily life is related to geographic locations and attributes and spatial distributions [28]. There is a large amount of geographic information in the food sampling quality and safety dataset. However, the geographic information of manufacturers and distributors in the dataset has not been fully used. Lam et al. [29] propose that the key words for the future of China’s food safety should include responsibility, accountability, and trackability. However, the geographical trackability depends on the full spatialization of the food production and sales chain. In order to make full use of the geographic information, this study employs social network analysis methods [30] to build multi-level networks of food production and sales, and analyzes the regional geographic dissemination characteristics of substandard foods. In China, social network analysis theories and methods are mainly applied to the researches on supply chain modeling of a certain product and the spread of its network risks [31, 32]. These studies do not consider the spatial location and spatial association of each node in the network, nor can they discover the regional characteristics of the food production and sales chain. Without cutting off the potential regional economic chains, it will be difficult to fundamentally control substandard foods production and sales driven by economic benefits. This study attempts to explore the regional dissemination risks of substandard foods from the circulation market, using social network analysis methods to identify important network characteristics, such as key cities and routes. According to the circulation characteristics of each region, we recommend governments formulating prevention strategies based on the local conditions to minimize the circulation of substandard foods.

In summary, from the research perspective, this study introduces geospatial concepts and social network analysis methods into food quality research, and expands the connotation and research methods of food quality research. From the data application perspective, the application of current food sampling quality and safety dataset still stays at the data-managed level. Due to the lack of effective means of data intelligent analysis, the value of the dataset has not been fully utilized. From the technical perspective, this study applies social network analysis methods to explore regional characteristics of multi-level circulation networks of substandard foods, which realizes the spatial transformation of food quality information and provides new perspectives for food quality analysis and supervision.

## 2. Materials and methods

### 2.1. Data collection and preprocessing

The database utilized in our work is the Food Safety Sampling Inspection Result Query System (https://spcjsac.gsxt.gov.cn/), which has published all the qualified and substandard food safety sampling inspection information since 2014. The system is managed by the State Administration for Market Regulation, and the data information is authoritative, comprehensive and reliable. We crawl all the data published on the website before July 9, 2019. There are 979,352 cases of the raw database, including 32,173 cases of substandard foods data. We eliminate cases that do not contain manufacturers and distributors information and filter the duplicated cases. After data preprocessing, 21,003 cases of substandard foods data are finally retained.

**Fig 1:**
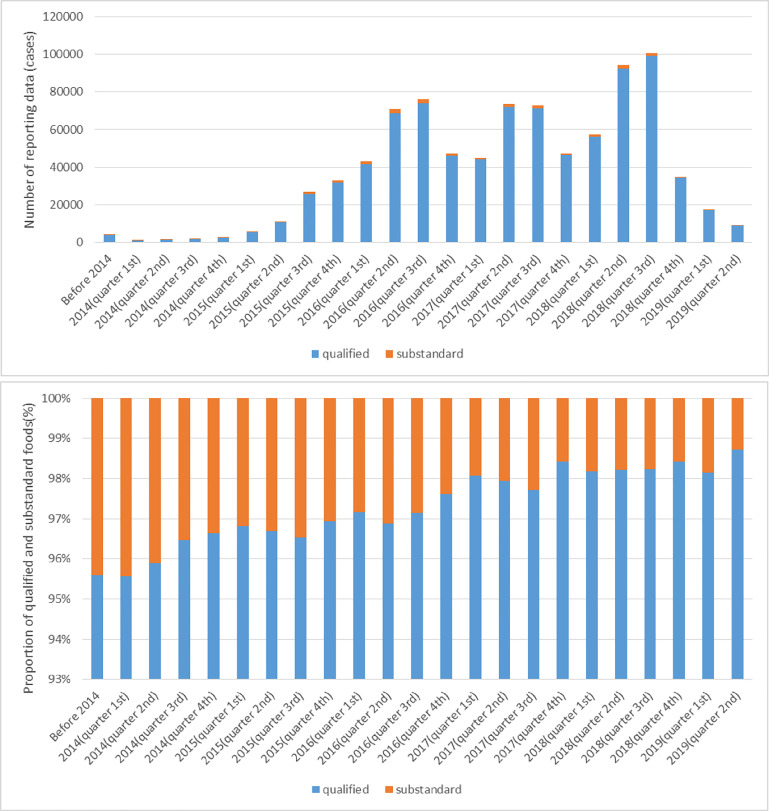
Quarterly statistics and analysis of foods data. (a) Number of reporting data. (b) Proportion of qualified and substandard foods.

Since we collected data before July 2019, many sampling inspection results of 2019 have not been uploaded to the system timely. Through the quarterly statistics and analysis of the website data, it can be found that from 2014 to 2018, the number of sampling inspection has been increasing year by year (Fig 1(A)). And on a quarterly basis, the proportion of substandard foods in sampling inspection results generally shows a downward trend (Fig 1(B)).

Sampling inspection is the most important and mature method for market supervision in China. China has implemented relevant food safety laws and regulations to standardize the sampling inspection work, mainly including the Food Safety Law of the People’s Republic of China and the Food Safety Sampling Inspection and Management Measures. In this study, we only use substandard foods data instead of all sampling inspection data. We assume that the sampling inspection is effective and can represent the current food safety situation, and we believe that the country will sample as fairly as possible in order to fully control China’s food safety situation.

### 2.2. Methods

#### 2.2.1. Latitude and longitude extraction

**Fig 2:**
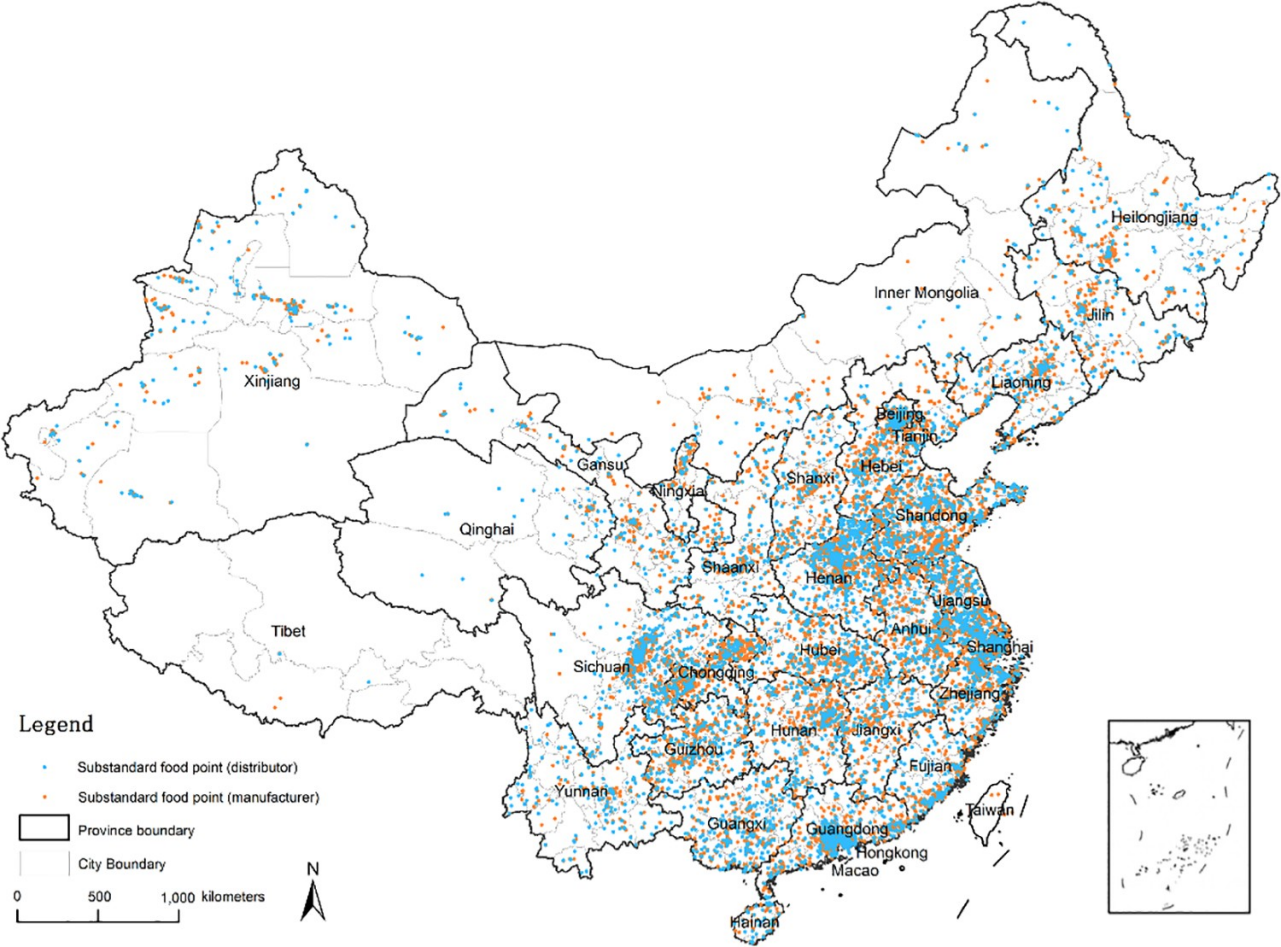
Substandard foods data.

The raw data only contains the structured addresses of manufacturers and distributors. For the subsequent social network analysis, we need obtain the latitude and longitude coordinates from the detailed structured addresses. In order to achieve this goal, we use the address resolution function of Geocoding API provided by Baidu Maps (http://lbsyun.baidu.com/index.php?title=webapi/guide/webservice-geocoding). For example, the address resolution result of “No. 445, Financial Road, Jiexiu City, Jinzhong City, Shanxi Province” is “lng:111.9083384, lat:37.0318653”. And the final spatial distributions of all substandard foods are shown in Fig 2.

#### 2.2.2. Social network analysis

This study constructs a province-level network and a city-level network to explore the regularities of substandard foods’ production and sales networks in China, utilizing Gephi [33], R and Key Player software packages [34]. In our work, a node denotes a province or a city, and a path represents a circulation route. The reason why we construct these two levels of networks is that they are consistent with China’s two main hierarchies of government. In this way, the analysis results can be combined with the hierarchy of government to develop preventive strategies that can be implemented at the existing administrative infrastructure. The indicators and methods used in this study are as follows.

(1) Statistical indicators

**Degree centrality.** Degree centrality is defined as the number of edges incident on a node. In a directed network, the direction of the edge also needs to be considered. Therefore, the degree of a node includes two categories, namely in-degree and out-degree. In-degree denotes the number of nodes pointing to a certain node, while out-degree represents the number of nodes that a certain node points to. In this way, a node’s degree is defined as the sum of its in-degree and its out-degree.

Both the degree and the weighted degree are used to measure the activity of a node participating in the substandard foods circulation in our work. And we also use the ratio between in-degree and out-degree to describe the circulation property.

$$d_{i}=\sum_{j}p_{ij},$$ (1)

$$\omega d_{i}=\sum_{j}\omega_{ij}p_{ij},$$ (2)

$$r_{i}=\frac{d_{i\_in}}{d_{i\_out}},$$ (3)

where, *d*_*i*_ represents the degree of node *i*, *ωd*_*i*_ represents its weighted degree, *r*_*i*_ represents its circulation structure, *p*_*ij*_ is the edge from *i* to *j*, *ω*_*ij*_ is the weight of the edge from *i* to *j*, *d*_*i*_*in*_ is the in-degree of node *i*, *d*_*i*_*out*_ is the out-degree of node *i*.

**Density.** Density refers to the closeness of connections between nodes in a network. The more edges between nodes of a fixed scale, the greater the density of the network. In a directed network, the density is represented by the ratio of the actual number of edges to the number of edges that are most likely to exist.

$$Density=\frac{e}{n\times (n-1)},$$ (4)

where, *e* represents the number of edges, and *n* represents the number of nodes. The density value is between 0 and 1. The closer the value is to 1, the closer the relationship between nodes is. It reflects the active degree that a node participates in the communication.

In this study, the calculations of the above statistical indicators are completed by Gephi.

(2) Node betweenness and edge betweenness

Degree centrality of a node depicts the local centrality of the node, measuring the transaction ability of the node itself, without considering whether it can control the others. Betweenness centrality of a node studies the degree of node controlling over resources. If a node is on the shortest paths of many other nodes pairs, the node has a high betweenness centrality and acts as a communicating bridge. Betweenness centrality is an important global geometric quantity which can reflect the function of the corresponding node or edge in the entire network. In the substandard foods circulation network, the distribution characteristics of the betweenness centrality reflect the position of different nodes and paths in the circulation relationships, and have a certain early warning effect on the prevention and control of the substandard foods transmission.

$$nb_{k}=\sum_{i}\sum_{j}\frac{p_{ikj}}{p_{ij}},i\neq j\neq k,$$ (5)

$$eb_{m}=\sum_{i}\sum_{j}\frac{p_{imj}}{p_{ij}},i\neq j,$$ (6)

where, *nb*_*k*_ denotes the betweeness centrality of node *k*, and the shortest paths number that node *k* is on the shortest path between *i* and *j* is expressed as *p*_*ikj*_, *eb*_*m*_ denotes the betweeness centrality of edge *m*, and *p*_*imj*_ represents the shortest paths number that edge *m* is on the shortest path between *i* and *j*.

In this study, node betweeness [35] and edge betweenness [36] are calculated by igraph package in R.

(3) Community detection–the Louvain algorithm

The Louvain algorithm is a community detection method on base of multi-level optimization modularity, and it is also a built-in algorithm in Gephi [37]. The advantage of this algorithm is that it is efficient and accurate. Lancichinetti and Fortunato believe it is one of the best performing community detection methods [38]. The modularity function is an optimization function which can describe the closeness of the discovered community. If a node joins a certain community and the modularity of the community is maximized, then the node should belong to the community. Otherwise, the node continues to stay in its current community.

The modularity is defined as follows:

$$Q=\frac{1}{2m}\sum_{i,j}\left[A_{ij}-\frac{k_{i}k_{j}}{2m}\right]\delta \left(c_{i},c_{j}\right),$$ (7)

where, *m* is the number of paths, *A* is the adjacency matrix. If *c*_*i*_ and *c*_*j*_ are the same, then *δ*(*c*_*i*_,*c*_*j*_) = 1, otherwise 0.

If the community where the current node is located only includes itself, the Louvain algorithm employs a technique to speed up the calculation when computing modularity’s increase of adding it to other communities.

$$\Delta Q=\left[\frac{\Sigma_{in}+k_{i,in}}{2m}-{\left(\frac{\Sigma_{tot}+k_{i}}{2m}\right)}^{2}\right]-\left[\frac{\Sigma_{in}}{2m}-{\left(\frac{\Sigma_{tot}}{2m}\right)}^{2}-{\left(\frac{k_{i}}{2m}\right)}^{2}\right],$$ (8)

where, Σ_*in*_ denotes the number of paths in the community, Σ_*tot*_ represents the number of paths incident on nodes of the community, *k*_*i*,*in*_ is the number of paths from node *i* to nodes of the community, *k*_*i*_ is the number of paths incident on node *i*.

The Louvain algorithm contains two steps. First, it traverses the nodes in the network and add a node to a community which could make the community’s modularity upgrade the largest, until it no longer changes. Second, the network is reconstructed by merging the existing communities into a few super nodes. The new edge weight is the sum of the edge weights of all the original nodes in the two super nodes. These two steps should be iterated until the algorithm becomes stable.

Community detection can reveal and quantify the clustering structures of substandard foods circulation network. By analyzing the relationships between circulation clusters and geospatial clusters, this study discusses the characteristics of food industry in different clusters and excavates different substandard foods circulation patterns.

(4) Key cities identification–the Borgatti’s method

The Borgatti’s method recognizes the key nodes set in a network which is maximally linked to all the others. Information can be optimally diffused by the network through treating key nodes as the seeds [34]. The method is useful to solve security issues, for example, in crime prevention, by taking a few people as seeds could identify an undercover crime network. In this method, the distance-weighted reach measure is defined as the sum of the reciprocal distances from the key nodes set *S* to all nodes, where the distance from *S* to a node is measured by the shortest distance. It maximizes the weights of nodes that are not belongs to *S*.

$$D_{R}=\frac{\sum_{i}\frac{1}{d_{Si}}}{n},$$ (9)

where, *D*_*R*_ represents the reciprocal distance indicator, *n* represents the total number of nodes, *D*_*Si*_ represents the shortest distance from *S* to node *i*.

In this study, we use this method to find key cities for substandard foods circulation, so as to conduct precise governance on these cities and to destroy the circulation network to the greatest extent. The key cities are detected by Borgatti’s free software named the Key Player Program (version 1.10).

## 3. Experimental results

### 3.1. Statistical characteristics

In this study, there are 34 provinces and regions and 361 cities participating in the circulation of substandard foods. All provinces and cities are connected to other nodes, and there is no province or city that is alone.

In the province-level network, an average of 196.529 substandard foods are circulated from each province to an average of another 18.235 provinces, which indicates that the network density is high (0.533). The city-level network has a larger scale, with 361 nodes and a network diameter of 6. But the network density of the province-level network is 15 times that of the city-level network (0.036). An average of 58.097 substandard foods are circulated from each city to an average of another 12.912 cities. However, for the province-level network, the distribution of substandard foods numbers circulated in and out of the provinces is highly skewed to the right, also called the long tail distribution. As shown in Table 1, the median of in-degree and out-degree are bigger than the mean of degree namely, a large number of substandard foods’ circulations are clustered on the left side of the distribution [39]. The circulation gap between provinces is large, and the numbers of substandard foods circulation in most provinces are small.

**Table 1:** Statistical indicators of the networks.

Statistical indicator	Province-level network	City-level network	Description
Size	34	361	The number of nodes.
Network diameter	3	6	The longest network distance between any pair of nodes.
Density	0.533	0.036	The proportion of the actual number of edges to the total number of edges that are most likely to exist.
Components	1	1	The number of separate parts.
Mean of weighted degree	196.529	58.097	On average, the number of substandard foods discovered per node.
Mean of degree	18.235	12.912	On average, the circulation paths that a node participates in.
Median (range) of weighted in-degree	147.5(0,895)	39 (0,640)	The number of substandard foods circulating into a node (median and range).
Median (range) of weighted out-degree	98.5 (1,756)	38 (0,787)	The number of substandard foods circulating out from a node (median and range).
Median (range) of in-degree	19(0,30)	10 (0,76)	The number of substandard foods circulating from other nodes into a node (median and range).
Median (range) of out-degree	19 (1,29)	8 (0,92)	The number of substandard foods circulating from a node to other nodes (median and range).

### 3.2. Circulation characteristics

**Fig 3:**
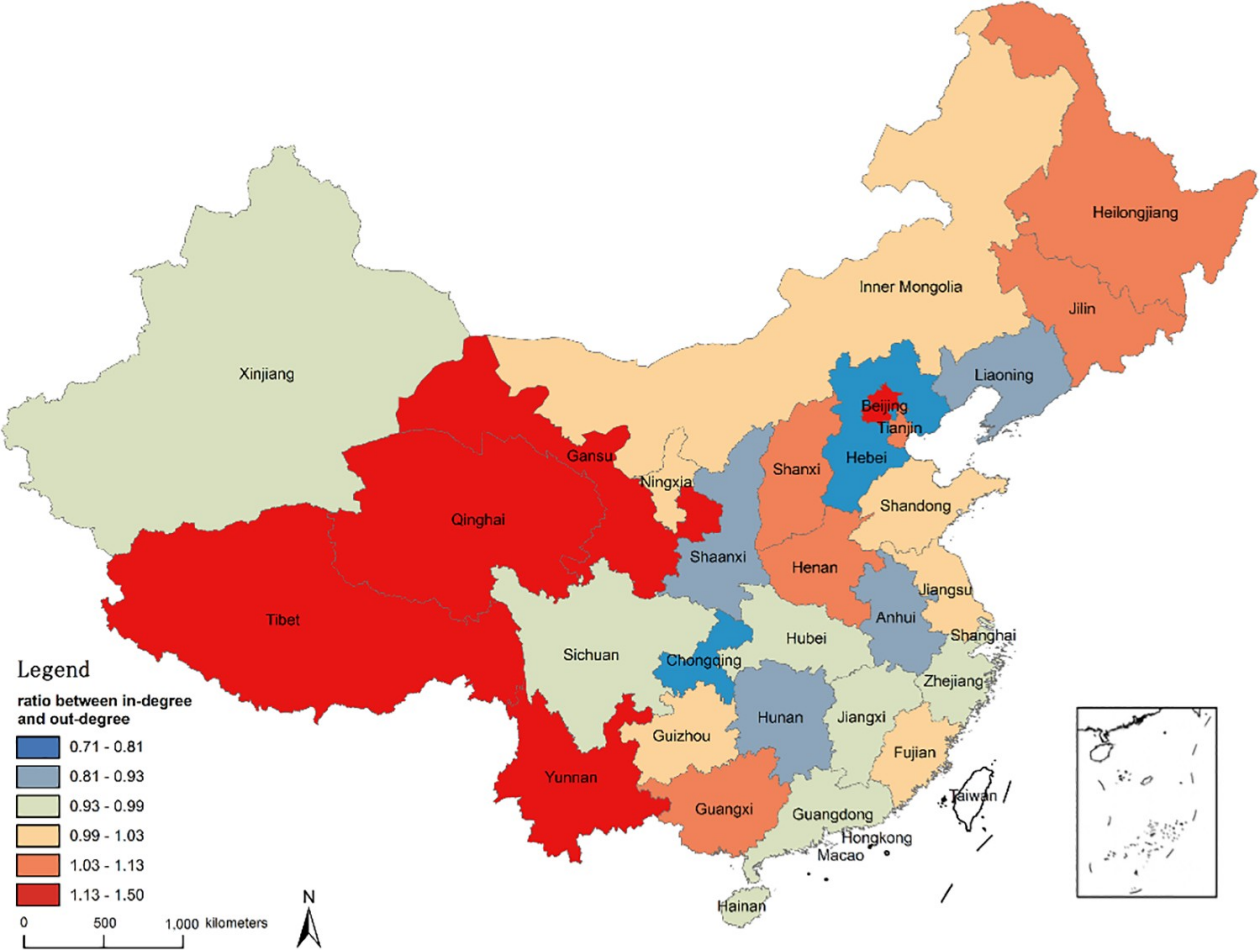
Ratio between in-degree and out-degree of 34 provinces and regions.

For analyzing the circulation characteristics of each province, the ratio between in-degree and out-degree is calculated first. As can be seen from Fig 3, 16 provinces are net destinations (i.e. ratio >1). In descending order, they are Tibet, Beijing, Qinghai, Yunnan, Gansu, Heilongjiang, Henan, Jilin, Guangxi, Tianjin, Shanxi, Jiangsu, Inner Mongolia, Shandong, Fujian, Ningxia. Other provinces are net sources, except for Guizhou where the ratio is equal to 1.

**Fig 4:**
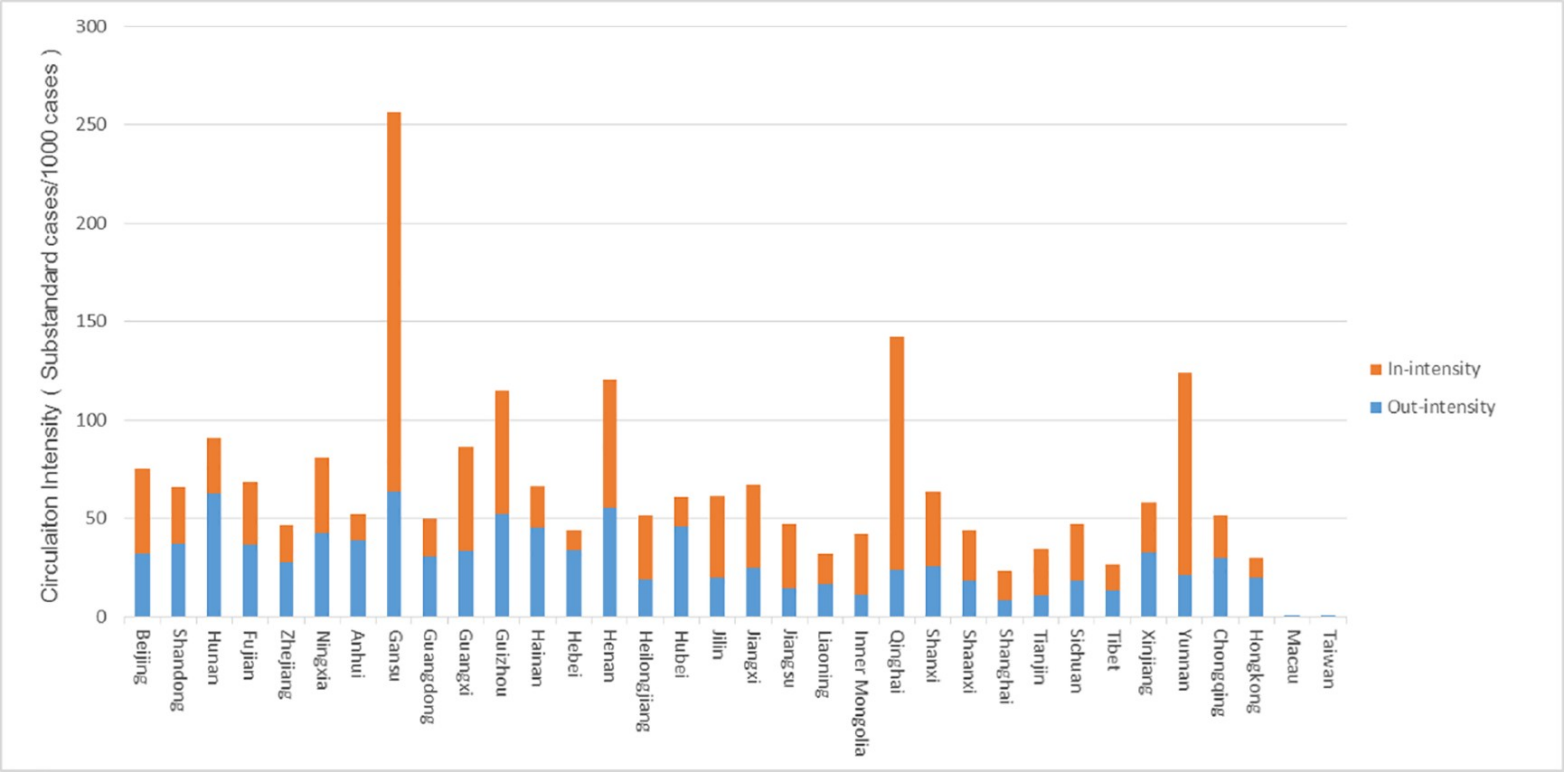
Circulation intensity of 34 provinces and regions.

In order to explore the circulation rates of substandard foods in 34 provinces and regions, we define the ratio of in-degree and out-degree to the sum of qualified and substandard foods as the circulation intensity. The results are shown in Fig 4. From the supply perspective, the provinces with higher out-intensity are Gansu, Hunan, Henan, Guizhou, Hubei, and Hainan. More than 42 substandard cases can be found for every 1,000 cases. From the demand perspective, the provinces with higher in-intensity are Gansu, Qinghai, Yunnan, Henan and Guizhou. The discovery rate of substandard foods is as high as at least 65 cases per 1,000 cases. These provinces should therefore be recognized as the priority locations for implementation of regulation interventions.

**Fig 5:**
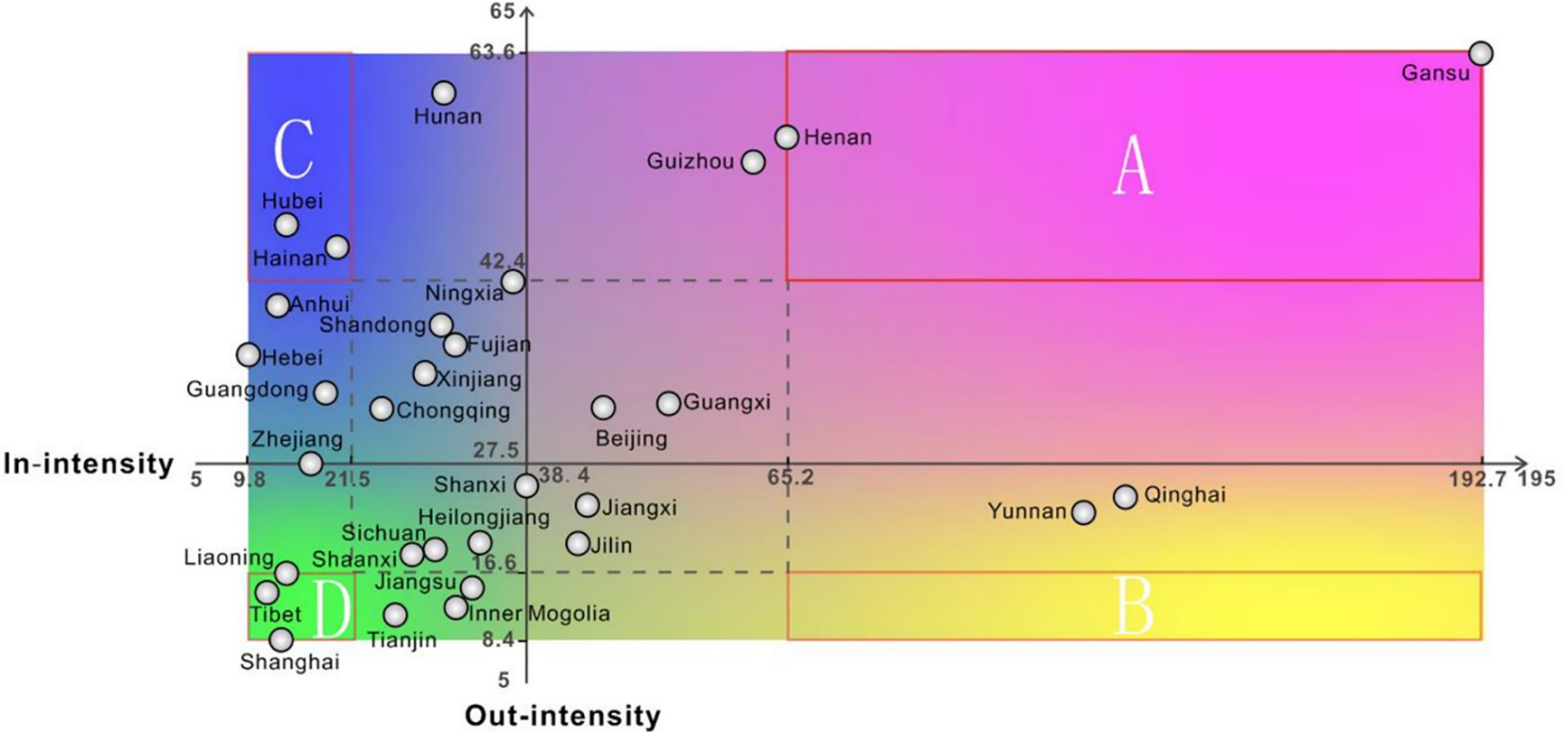
Circulation patterns of 34 provinces and regions. (A. Higher circulation intensity: Henan, Gansu; B. Net destination: None; C. Net source: Hainan, Hubei; D. Lower circulation intensity: Liaoning, Tibet, Shanghai).

The in-intensity and out-intensity of 34 provinces and regions are classified according to the natural breakpoint method. Based on this, we explore the substandard foods’ circulation patterns of various provinces. The classification standards are exhibited in Table 2, and the classification results are shown in Fig 5. It can be found that Henan and Gansu have both higher in-intensity and higher out-intensity, so the circulation risks of these two provinces are also the highest. When food safety supervision is carried out, it is necessary not only to strengthen the supervision of food production and processing enterprises in the province, but also to strictly monitor the food quality circulated inside from the other provinces. Hainan and Hubei have higher out-intensity but lower in-intensity, and it is necessary to strengthen supervision of food production and processing enterprises in their provinces and to prevent the production and export of low-quality foods.

**Table 2:** Classification standards of provinces’ circulation patterns.

Indicator	Lower	Low	High	Higher
In-intensity	[9.8–21.1)	[21.1,38.4)	[38.4,65.2)	[65.2,192.7]
Out-intensity	[8.4–16.6)	[16.6,27.5)	[27.5,42.4)	[42.4,63.6]

Through statistical analysis, we find that most of the substandard foods are circulated in the same province, accounting for 81.7% of all routes. 62.5% of the sources of substandard foods circulating and 43.6% of the destinations of substandard foods circulating are clustered in 8 provinces. In descending order, they are Henan, Shandong, Guangdong, Fujian, Hebei, Zhejiang, Hunan and Hubei. These cities are located in eastern and central China. And this is related to the geographical distributions of food production and processing enterprises across the country. The eastern region accounts for 42.1%, the central for 26.8%, the western for 18.9%, and the northeast for 12.2% [40]. Except for Guangdong, most of the inter-provincial circulation paths are between neighboring provinces, within short distances. Similar to the province-level network, substandard foods are mostly circulated inside the same city, accounting for 57.2% of all routes, of which the provincial capitals account for 23.2%. The top 10 cities in descending order are Chonqing, Beijing, Shanghai, Changsha, Zhengzhou, Wuhan, Qingdao, Linyi, Nanning and Shenyang. As shown in Table 3, from the city-level network perspective, substandard foods are mostly exported from prefecture cities to provincial capitals.

**Table 3:** Top 10 circulation paths (according to the number of circulation paths).

	Top 10 circulation paths (in descending order)
Province-level network	Shandong->Henan, Chongqing->Guangdong, Henan->Shandong, Guangdong->Henan, Hunan->Guangdong, Hebei->Shandong, Anhui->Henan, Hebei->Henan, Hebei->Beijing, Henan->Jiangsu.
City-level network	Zhangzhou->Xiamen, Jieyang->Guangzhou, Dongguan->Guangzhou, Chongqing->Guangzhou, Jingzhou->Wuhan, Kaifeng->Zhengzhou, Zhangzhou->Fuzhou, Bozhou->Hefei, Foshan->Guangzhou, Jiaozuo->Zhengzhou.

### 3.3. Substandard foods circulation cluster of cities

The highly networked spatial connection is the key element to form the city cluster. The cluster with relatively higher cohesion has closer economic and social ties and has a higher degree of centrality. Fig 5 shows the 13 clusters of 361 cities identified by the Louvain algorithm. The obvious regional clusters indicate that there are differences in the circulation characteristics of substandard foods. It is worth noting that there are connections between clusters, and some clusters are not consistent with geographical distributions. Specifically, Chongqing, as a city in the southwest, is considered to have a unified pattern (cluster 4) with southeastern coastal cities in Guangdong Province. Both the import number and export number of substandard foods in this cluster are much higher than the other cities. Cities belonging to Jiangxi Province and Hainan Province are not geographically adjacent, but they exhibit strong network relevances (cluster 7). The city with the highest node betweenness in a cluster is usually closely connected to cities in other clusters, which indicates that it is the main network gatekeeper. They are Chongqing, Beijing, Zhengzhou, Shanghai, Guangzhou, Changsha, Wuhan, Linyi and Hefei. With the exception of Linyi in Shandong Province, these cities are almost provincial capitals, indicating cities with better economic conditions and convenient transport always serve as centers of the circulation of substandard foods.

#### 3.4. Key cities

The refined analysis in the city-level network is helpful to discover the problems behind the substandard foods circulation network. We detect the cities which have the largest number of substandard foods circulations. First, we identify the main cities participating in the circulation of substandard foods according to the indicator of degree centrality, and the results are shown in Fig 7. In terms of weighted degree, Chongqing has the highest circulation rate of substandard foods (1,427, accounting for 3.39% of the total), with both high import and export. For the next 20 cities, these numbers dropped rapidly, from 825 to 303 cases (from 1.96% to 0.70%). Chongqing, Beijing, Zhengzhou, Shanghai, and Guangzhou each have more than 600 cases (10.07%), followed by Changsha, Wuhan, Linyi, and Hefei, each with more than 400 cases (4.67%).

**Fig 6:**
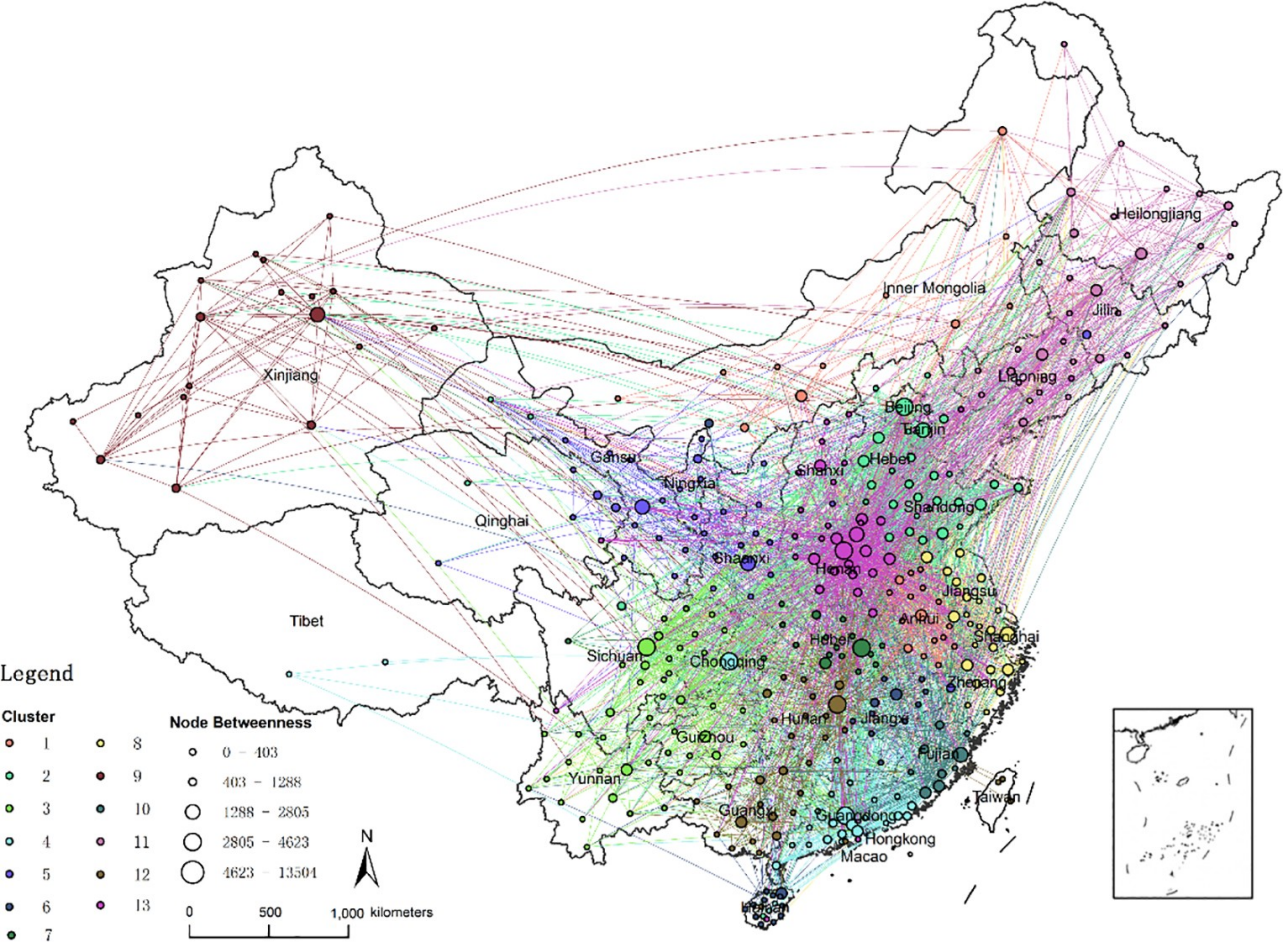
Substandard foods circulation cluster of cities.

**Fig 7:**
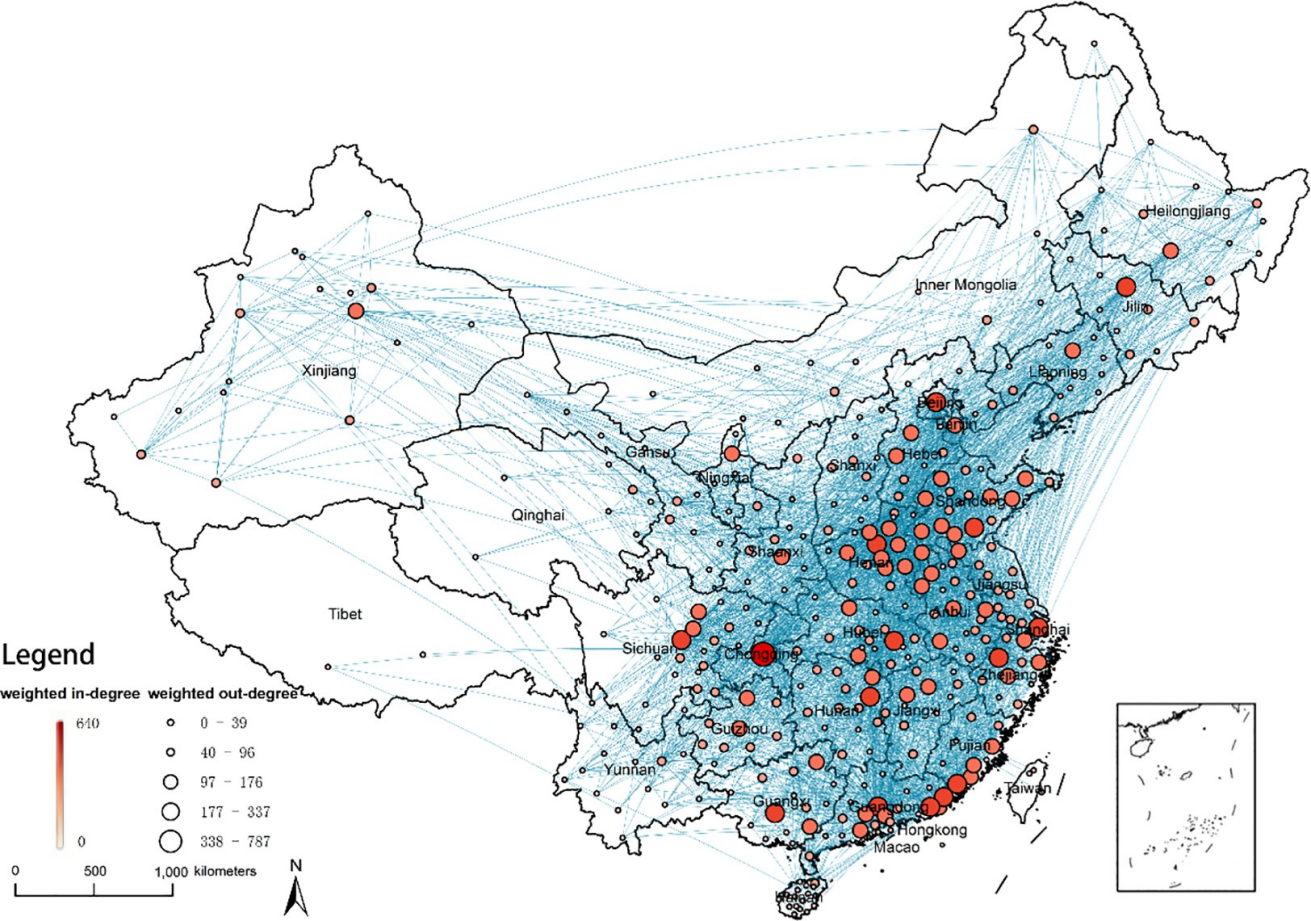
Circulation patterns of 361 cities. (The lines indicate circulation routes. The size of the circle represents the weighted out-degree of each city. The color of the circle represents the weighted in-degree of each city.).

In this study, we also identify key cities and paths by calculating the indicator of betweenness centrality. Betweenness centrality can measure the global influence and function of a city or a path in the whole circulation network. All the reported substandard foods’ circulation paths’ lengths are only 1, missing the intermediate information. We assume that the quality of foods will change during the circulation process, and this change will be transmitted through the circulation networks. Under this assumption, we apply the node betweenness and edge betweenness to find the key intermediate nodes and paths. As displayed in Fig 6, Beijing, Zhengzhou, Chongqing, Changsha, Chengdu and Guangzhou have the greatest impacts on the circulation of substandard foods, with node betweenness values ranging from 6.24% to 2.95%. These cities are all provincial capitals. According to the results shown in Fig 6 and Fig 7, underdeveloped regions always have less impacts on the network, including the northeast, northwest and southwest regions. Next, we calculate edge betweenness to determine the key intermediate paths, and the top 10 paths are listed in Table 4. These 10 paths control 34.98% of the total paths. These paths mainly originate from the provincial capitals, for example, Zhengzhou, Beijing and Changsha.

**Table 4:** Top 10 key paths (according to the edge betweenness).

Key paths	Edge betweenness(%)
**Zhengzhou(Henan)-Urumqi(Xinjiang)**	5.51
**Wuhai(Inner Mongolia)- Hohhot(Inner Mongolia)**	4.97
**Beijing-Chongqing**	3.98
**Beijing- Hohhot(Inner Mongolia)**	3.49
**Dongfang(Hainan)-Lianyungang(Jiangsu)**	3.18
**Ordos(Inner Mongolia)-Zhengzhou(Henan)**	2.87
**Changsha(Hunan)-Yueyang(Hunan)**	2.86
**Shanghai-Jinzhou(Liaoning)**	2.75
**Haikou(Hainan)-Zhangzhou(Fujian)**	2.70
**Beijing-Wuhan(Hubei)**	2.67

In order to control and prevent the circulation of substandard foods to the greatest extent from the perspective of information diffusing, we employ the Borgatti’s method to identify the set of key cities, thereby formulating more precise policies to combat substandard foods’ circulation. Through strengthening the supervision of relevant cities, government can investigate deep-seated substandard foods’ production and sales chains. Table 5 shows the percentage of nodes reached by each key city set according to the reciprocal distance index.

**Table 5:** Key cities based on information diffusing.

*D*_*R*_ (%)	56.9	64.8	70	73.9	76.8	79.2	81.3	83	84.6	86
Chongqing	**○**	**○**	**○**	**○**	**○**	**○**	**○**	**○**	**○**	**○**
Beijing		**○**	**○**	**○**	**○**	**○**	**○**	**○**	**○**	**○**
Zhengzhou			**○**	**○**	**○**	**○**	**○**	**○**	**○**	**○**
Chengdu				**○**	**○**	**○**	**○**	**○**	**○**	**○**
Tianjin					**○**	**○**	**○**	**○**		
Jieyang						**○**		**○**	**○**	**○**
Changsha							**○**	**○**	**○**	**○**
Fuzhou							**○**			
Anyang								**○**		
Shanghai									**○**	**○**
Deyang									**○**	**○**
Shijiazhuang									**○**	**○**
Xinxiang										**○**

Although there are 361 cities involved in the production and sales of substandard foods, but we only need to lock the target in 3 cities to connect more than 70% of the network. Chongqing, which has the highest frequency, can reach nearly 57% of the network directly. If we use diverse set sizes, other key cities except Chongqing can be detected, including Beijing, Zhengzhou and Chengdu. There are certain overlaps between key cities detected by information diffusing and the key cities determined by node betweenness. This is because these two indicators are both designed to identify major nodes through which a large number of paths flow. It can be seen from Table 6 that Xinxiang, Deyang, and Shijiazhuang have not been identified by the previous indicators, but they are important from the perspective of information diffusing. As the total number of selected key cities increases, the reciprocal distance index also increases. Nonetheless, only when 49 key cities are included, all cities in the network can be covered.

**Table 6:** Summary of the key cities identified by various indicators.

Indicator	Top 10 key cities
Weighted degree	Chongqing, Beijing, Zhengzhou, Shanghai, Guangzhou, Changsha, Wuhan, Linyi, Hefei, Hangzhou
Weighted in-degree	Chongqing, Beijing, Zhengzhou, Guangzhou, Shanghai, Wuhan, Changsha, Hefei, Ningbo, Linyi
Weighted out-degree	Chongqing, Shanghai, Beijing, Zhengzhou, Changsha, Jieyang, Wuhan, Chaozhou, Zhangzhou, Linyi
Node Betweenness	Beijing, Zhengzhou, Chongqing, Changsha, Chengdu, Guangzhou, Wuhan, Urumqi, Xi’an, Shanghai
Information diffusing (reciprocal distance index = 86%)	Chongqing, Beijing, Zhengzhou, Chengdu, Shanghai, Xinxiang, Deyang, Changsha, Jieyang, Shijiazhuang

## 4. Discussion

In China, the food industry is dominated by small-size and medium-size enterprises, food safety issues are constantly emerging and the cost of supervision is very high. Food circulation is an important part of the entire food chain. Because of the nature of foods, and trends of food production, processing or consumption in other places, the factors affecting the quality and safety of foods in the field of circulation have increased. The pass rate of foods sampling inspection in the circulation part is increasing gradually, but problems are still emerging in an endless stream [41].

This study carries out a series of analyses on the networks of substandard foods production and sales. Although the circulation density of substandard foods is relatively dense at the provincial level (mainly concentrated in a few provinces), it is sparse and highly diffused at the municipal level. Despite the circulation of substandard foods is concentrated in some provinces, the circulation within and between provinces is relatively scattered. Such characteristics also increase the difficulty of substandard foods’ supervision. It is urgent to study the in-depth regional characteristics and to identify key cities of the production and sales of substandard foods, thereby formulating targeted strategies to break the circulation network according to local conditions and improving the efficiency of substandard foods’ supervision.

Because the circulation of substandard foods is mostly local circulation and short distance trafficking, many problems are difficult to find and easier to cover up. We propose to publicize and encourage the public participating in food safety supervision in key cities. In addition, a platform should be established to facilitate the public reporting violations of substandard foods’ production and sales.

This study constructs city clusters according to the network connection characteristics of substandard foods’ production and sales. Specific to the core cities in different clusters, government can design corresponding supervision schemes. The successful experience of governance in the core city can be directly applied to other cities in the same cluster to reduce the regulatory costs. The government can change the cluster from large to small through effective supervision and cut off the circulation chain.

According to the results of key cities identification, underdeveloped regions always have less impacts on the network, including the northeast, northwest and southwest regions. And key paths always originate from provincial capitals. It is worth noting that Chongqing, Beijing, Zhengzhou and Changsha are identified as key cities according to all indicators in this study. At least four indicators can identify Shanghai and Wuhan. Clearly, the importance of these cities makes them a top priority in combating substandard foods’ circulation networks.

## 5. Conclusions

This study applies GIS and social network analysis methods to deeply explore the regional circulation characteristics of substandard foods. Based on GIS method, the dataset of Food Safety Sampling Inspection Result Query System is used to obtain the geographical locations of substandard foods’ manufacturers and distributors. Then the province-level and city-level substandard foods’ circulation networks are constructed and social network analysis methods are applied to explore the characteristics of the circulation networks, aiming at detecting the key cities and paths of substandard foods’ circulations. The experimental results show that the circulations of substandard foods are characterized by dense province-level network and sparse city-level network. The circulation paths of substandard foods are mostly local and short-distance. According to the network connection characteristics and indicator calculation results, 361 cities are divided into 13 city clusters and Chongqing, Beijing, Zhengzhou, Changsha, Shanghai and Wuhan are identified as key cities.

There are some limitations in this research. First, there is the limitation of data quality. The data we used lacks some detail information. For example, the intermediate cities along the circulation routes are not listed. With this detailed information, we can design preventive measures more precisely. The results in this study may deviate from the actual situations due to the limitation of data quality. The regional imbalances in the circulation of substandard foods suggest that the regulation in underdeveloped regions may be weak. Second, this study only uses the food sampling quality and safety dataset for analysis. In the subsequent study, we will try to integrate other auxiliary data (such as socio-economic population data that can reflect spatial heterogeneity, accessibility of transportation networks, etc.) to quantify the circulation characteristics, so as to further explore the patterns and causes of substandard foods’ circulation.

## Supporting information

S1 File (XLS)
